# Psychological Adjustment of Children and Adolescents with 22q11.2 Deletion Syndrome and Their Mothers’ Stress and Coping—A Longitudinal Study

**DOI:** 10.3390/ijerph18052707

**Published:** 2021-03-08

**Authors:** Wolfgang Briegel, Christoph Andritschky

**Affiliations:** 1 Department of Child and Adolescent Psychiatry, Psychosomatics and Psychotherapy, Leopoldina Hospital, 97422 Schweinfurt, Germany; 2 Department of Child and Adolescent Psychiatry, Psychosomatics and Psychotherapy, University of Würzburg, 97080 Würzburg, Germany; christoph.andritschky@wz-kliniken.de; 3 Department of Pneumology, Critical Care and Allergology, Lung Centre South-West, 88239 Wangen im Allgäu, Germany

**Keywords:** 22q11.2 deletion syndrome, behavior problems, coping strategies, longitudinal study, maternal stress, satisfaction with life

## Abstract

At present, there is a lack of longitudinal studies on the psychological adjustment of both children and adolescents with 22q11.2 deletion syndrome (22q11.2DS) and their primary caregivers. To fill this gap, we performed a four-year follow-up study. Mothers filled out the Child Behavior Checklist 4–18, the Social Orientation of Parents with Handicapped Children questionnaire to assess maternal stress and coping strategies, and the Freiburger Personality Inventory-Revised—subscales strain and life satisfaction. Fifty-five subjects with 22q11.2DS (26 males and 29 females; age: M = 10.79 years, SD = 3.56 years) and their biological mothers (age: M = 40.84 years, SD = 4.68 years) were included in this study. Significantly higher levels of behavior problems than in the general population and an increase in these problems, especially internalizing ones, over time could be found. In contrast, maternal stress did not change significantly over time, but mothers demonstrated increased levels of strain and reduced life satisfaction at T2. Thus, careful monitoring as well as early and adequate interventions, if indicated, should be offered to families with a child with 22q11.2DS, not only for somatic complaints but also for problems with psychological adjustment.

## 1. Introduction

22q11.2 deletion syndrome (22q11.2DS), a multi-systemic genetic disorder, is the most frequent microdeletion syndrome in humans with an estimated prevalence of 1 in 2000–4000 live births [1]. About 90% of affected individuals have a 3 Mb microdeletion, resulting in hemizygosity of about 106 genes, and 5–8% show a nested deletion of 1.5 Mb, causing haploinsufficiency of 30 coding genes [1,2]. The deletion is caused by a de novo mutation in 90–95% of cases [1]. Although some genes are well-described (e.g., T-Box1 Transcription Factor, Catechol-O-Methyl Transferase), haploinsufficiency of genes does not explain the heterogeneous penetrance and severity of clinical phenotypes [2]. Patients can show a variety of somatic symptoms: congenital heart defects; velopharyngeal dysfunction with or without cleft palate; immunodeficiency; hypocalcemia due to hypoparathyroidism, thymic hypo- or aplasia; and dysmorphic facial features [1]. 

In addition to physical health concerns, mental health problems are of high relevance among individuals with 22q11.2DS. Similar to somatic symptoms, neurodevelopmental/psychiatric phenotypic expression is highly variable, both inter-individual and intra-individual (during the course of development) [3,4]. However, typical changes in the behavioral/psychiatric phenotype could be found across age [3,4]. From infancy onward, developmental delay of psychomotor functions (often with hypotonia) and speech/language are very common [5,6]. The average Full-Scale IQ (FSIQ) is 70–75 with about 55% of individuals presenting with intellectual functioning in the borderline to normal range and about 45% having a mild to moderate intellectual disability (FSIQ < 70) [7,8]. Quite recently, a longitudinal study including 829 patients ages 8–24 years found that, overall, individuals with 22q11.2DS showed a decline of seven FSIQ points or nine Verbal IQ (VIQ) points, and that a significantly steeper VIQ decline preceded the onset of psychosis [9]. 

As children with developmental delay or intellectual disability (ID) are about three times as likely to score in the clinical range for emotional and behavioral problems than normally developing children [10,11,12], it is not astonishing that individuals with 22q11.2DS have been found to be at an increased risk for developing behavior problems and psychiatric disorders [3,4,13]. However, there seems to be a specific behavioral/psychiatric phenotype in 22q11.2DS [3,4]. With regard to the categorical approach of psychiatric diagnoses, individuals with 22q11.2DS show higher prevalence rates of autism spectrum disorder (ASD), attention-deficit/hyperactivity disorder (ADHD), mood/anxiety disorders and psychotic disorders when compared to individuals with idiopathic ID [4]. In contrast, the prevalence rates of both disruptive disorders and substance use disorders are lower than in the idiopathic ID population [4]. While autism spectrum disorder, attention-deficit/hyperactivity disorder and anxiety disorders have been found to be the most common diagnoses during childhood, prevalence rates of psychotic and mood disorders increase significantly later on in life [9] with about 5% of youth and up to 40% of adults over 25 years developing schizophrenia spectrum disorders [9,14]. 

In line with these findings, several cross-sectional studies which used a dimensional approach (by means of parent and teacher reports) have reported increased rates of clinical behavior problems: 25–60% of 4–18-year-old subjects with 22q11.2DS were rated clinical with predominantly social and attention problems [15,16] whereas slightly lower rates of clinical cases (24%) have been found among 1.5–3 year old children [17]. Moreover, young individuals with 22q11.2DS scored significantly higher for internalizing problems when compared to community controls, siblings, age and intelligence matched children with speech-language delays without a genetic cause or children with clefts [13,18,19,20]. Additionally, they presented with lower social skills than community controls and siblings [20]. Cross-sectional studies have suggested an increase in behavior problems, especially internalizing problems, with age [16,17]. Specifically, a positive correlation between the subject’s age and the CBCL/4–18 scales withdrawn, anxious depressed, thought problems, social problems, attention problems, internalizing problems and total problems as well as the CBCL/1.5–5 scale anxiety problems has been reported [16,17]. Regarding gender differences, one study reported higher scores for total problems and, surprisingly, internalizing problems in 4–18-year-old males compared to females [16] whereas other researchers did not find any gender differences [15]. 

It is well documented that the frequency of child behavior problems, especially externalizing problems, is significantly positively associated with parental stress which can be conceptualized as “a complex process in which adults feel overwhelmed in their role in relation to the responsibilities associated with it” [11,21,22,23]. A recent meta-analysis also found highest parenting stress levels for parents of children with ASD and developmental delay compared to parents of children from other clinical groups [23]. Some studies have shown that high levels of parenting stress contribute to a worsening in child behavior problems over time and vice versa, e.g., [11,24]. Moreover, this association seems to be more prominent among parents of children with mental and physical health problems [23]. Thus, regular assessment of parenting stress as part of routine care and throughout behavioral intervention programs has been recommended, especially for children at high risk for behavior problems and/or mood disorders, such as children with ASD and developmental delay or chronic illnesses (e.g., cancer, diabetes) [23]. 

Although primary caregivers of children and adolescents with 22qq11.2DS typically have to face many challenges due to their child’s genetic condition, studies on parental stress and coping strategies have been rare until now with longitudinal studies lacking completely. Cross-sectional studies have found low to average levels of parental stress when compared to other parents of mentally and/or physically disabled children (16,17). Additionally, parents of young subjects with the deletion did not differ from other parents of mentally and/or physically disabled children with regard to coping strategies [25]. In contrast, parental stress (as assessed with the Parenting Stress Index (PSI, [26]) and strain (as a personality aspect) were significantly higher among parents of children and adolescents with the deletion compared to age and gender matched subjects of the general population [16,20]. Moreover, a recent longitudinal study demonstrated that internalizing symptoms in children with 22q11.2DS predicted poor social functioning (e.g., problems with interpersonal relationships, social leisure activities) in young adulthood, and this effect was mediated by parenting stress [20]. Despite of these findings, primary caregivers of subjects with 22q11.2DS have been found to show levels of satisfaction with life similar to age and gender matched subjects of the general population, suggesting successful parental coping with stress [16,17]. No significant correlation of parental stress and strain with the child’s age has been reported so far [16,17]. However, parental stress and strain were found to be significantly positively correlated with child behavior problems [16,17] and handicap-related problems [25], i.e., the perception of problems in various areas of life due to the child’s disabilities. 

As there is a lack of longitudinal studies focusing on (i) mental health problems in young subjects with 22q11.2DS, (ii) their mothers’ stress and coping strategies, and (iii) mothers’ personality aspects, we wanted to study these important aspects by means of a four-year follow-up of the studies reported by Briegel et al. [16,17]. Based on the literature described above, we expected to find:

1. Significantly more behavior problems (reported on the CBCL/4–18) at Time 1 (T1) and Time 2 (T2) compared to the general population.
2. Significantly higher scores on CBCL global scales in boys compared to girls, both at T1 and T2.
3. A significant increase in clinical behavior problems over time (as reported on the CBCL, especially internalizing and total problems).
4. A significant increase in maternal stress over time.
5. Significantly higher scores of maternal stress at T2 for subjects suspicious of ASD compared to subjects without such a suspicion.
6. Significantly higher scores of maternal stress at T2 for subjects with ID compared to subjects without such a disability.
7. Significantly higher scores for maternal strain, but not for maternal satisfaction with life, at T2 when compared to age and gender-matched subjects of the general population.
8. A significant increase in the personality aspect strain, but no significant change of maternal satisfaction with life over time.
9. Significantly positive relations between maternal stress and strain and the CBCL global scales at both study times.
10. Maternal stress at T1 to have positive predictive value for child behavior problems as assessed with the CBCL/4–18 total problems scale at T2.

No a priori hypotheses regarding maternal coping strategies have been made. 

## 2. Materials and Methods

### 2.1. Design

An ethics committee approved the current study which followed the recommendations of the Declaration of Helsinki and was supported by the German 22q11.2DS foundation, KiDS 22q11. The executive board of KiDS 22q11 had been informed about the purpose and content of this follow-up in advance. A set of study materials was delivered through KiDS 22q11 to the mothers of all initially 1–14-year-old children and adolescents who could have been included in previous studies on child psychological adjustment, parental strain and life satisfaction (T1; [16,17]). All mothers were asked to fill out and to send back questionnaires anonymously by postal services. 

### 2.2. Sample

94 children and adolescents with 22q11.2DS aged 1–14 years could be included in previous studies on behavior problems, parental stress, coping strategies and personality aspects like strain and satisfaction with life (T1; for more details see [16,17]). At four years follow-up (T2), 57 primary caregivers sent back filled-out questionnaires (response rate: 60.64%). As study materials had been filled out by two fathers who were the primary caregivers at T2, these two cases had to be excluded from further analysis. Thus, the study sample comprises 55 subjects with 22q11.2DS (26 males and 29 females; age: *M* = 10.79 years, *SD* = 3.56 years, range: 5–18 years,) and their biological mothers (age: *M* = 40.84 years, *SD* = 4.68 years, range: 31–52 years) who participated in our study both at T1 and T2. In all cases, the mothers were the primary caregivers (no changes between T1 and T2).

In all patients, the deletion had been diagnosed by fluorescence in situ hybridization (FISH) and was due to a de novo mutation. According to their mothers’ information, 48/55 children (87.27%) had a congenital heart defect, 34/55 suffered from thymic malformations, and 27/54 (missing information: *n* = 1) from immune deficiency. 20 children (36.36%) had (had) cleft palate or velopharyngeal insufficiency, 14 (25.45%) had developed seizures, and 17 had shown hypocalcemia. At T2, 16 subjects had been seen by a child and adolescent psychiatrist for various reasons (three more than at T1), and two of them were treated with methylphenidate (versus none at T1). Additionally, three subjects had (had) behavior therapy at T2 (versus no subject at T1). 

At T2, only three children were too young to attend school compared to 33 at T1. Of the remaining 52 children and adolescents, 30 subjects attended a school specialized in providing education for children with special needs (including schools for physically disabled children, children with language or intellectual disabilities). At follow-up assessment, only 11 children were classified by their mothers as having at least average intelligence (compared to 19 at T1). Four mothers could not make a decision at T1 and T2, respectively. 

46/55 parents lived together (compared to 49 at T1), 7 mothers were single parents (vs. 6 at T1). All mothers had attended school for at least nine years, 10 had graduated from university. At T2, 25 mothers were housewife, retired or unemployed (compared to 26 at T1), the rest worked at least part time. At 2, 52/55 subjects with 22q11.2DS had at least one sibling (T1: 43/55), and four mothers rated the family’s financial situation as not sufficient (vs. one mother at T1). 

### 2.3. Assessment Tools

By post, all mothers received a set of materials which contained:

- A set of questions comprising personal, physical and psychosocial history of the subjects with 22q11.2DS.
- The well-validated German version of the Child Behavior Checklist (CBCL/4–18 [27,28]) for patients up to the age of 18 years. This parent-report measure consists of 113 items for parents of children aged 4–18 years (ratings: 0 = not true, 1 = somewhat or sometimes true, and 2 = very true or often true) Based on statistical groupings of sets of behaviors, the following eight symptom scales can be calculated: Social Withdrawal, Somatic Complaints, Anxiety/Depression, Social Problems, Thought Problems, Attention Problems, Delinquent Behavior, and Aggressive Behavior. Additionally, the CBCL/4–18 allows two broader groupings of syndromes: Internalizing Problems (combining the Social Withdrawal, Somatic Complaints, and Anxiety/Depression scales), and Externalizing Problems (combination of the Delinquent Behavior and Aggressive Behavior scales). The sum of all the problem items constitutes the Total Problems score. Results are given as *t*-values with clinical cases being defined as *t*-scores > 70 (for syndrome scales) or > 63 (for global scales).
- Subscales strain (tense, overwrought, stressed vs. unstrained, unpressured, able to handle stress) and life satisfaction (contented with life, optimistic, hopeful vs. discontented, depressed, negative attitude towards life) of the Freiburger Personality Inventory-Revised (FPI-R [29]), a well validated German personality measure. Mothers were asked to answer (true/not true) the 12 items of each scale. Results are given as stanine values with clinical cases being defined as stanine scores of nine (for strain), and one (for life satisfaction).
- The Social Orientation of Parents with Handicapped Children (SOEBEK [30]), a standardized and validated German questionnaire to assess coping strategies and stress among caregivers of 1–14-year-old subjects with mental and/or physical disability which should thus allow to take better account of the special situation of parents of children with 22q11.2 DS. The questionnaire comprises five scales: the four coping strategies partnership intensification (6 items), ability to meet own needs (5 items), use of social support (6 items) and focusing on the child with a disability (6 items) as well as the 20-item scale parental stress for which a good correlation with the total score of a preliminary German version of the PSI could be demonstrated [31]. While all items of the coping strategies are rated on a 6-point Likert scale from never (1) to very often (6), parental stress comprises 17 items to assess the frequency of different stressors from 1 (very seldom) to 5 (very often). Three further items are rated on two levels. There are different norms for mothers and fathers. Results are given as raw scores and percentiles for children with physical or physical and mental handicaps. Scores above the 95th percentile were defined as clinical for the scales parental stress and focusing on the child with a disability while scores below the 5th percentile were defined as clinical for the following coping strategies: partnership intensification, ability to meet own needs, and use of social support.
- The Behavior and Social Communication Questionnaire (VSK [32]), a well-validated German adaptation of the Autism Screening Questionnaire (ASQ [33]), was only given to mothers of subjects who had been younger than 6 years at T1. A cut-off of 17 has been found to have a specificity of 99% and a sensitivity of 92% [32].

### 2.4. Data Analysis

For statistical analysis of the results, we used SPSS 24.0 (IBM Corp., Armonk, NY, USA). Parametric tests were preferred for larger samples (*n* ≥ 30). Specifically, we performed the paired student *t*-test to analyze differences between T1 and T2, and the *t*-test for independent samples to analyze differences between groups. As we did not include a control group in this study, the one sample *t*-test was used to analyze differences between the study population and the general population. To measure the strength of the relationship between continuous variables, Pearson’s r was calculated. In case of small sample sizes (*n* < 30) the nonparametric Mann-Whitney U test was used to compare differences between T1 and T2. Effect sizes were calculated and interpreted following Cohen’s suggestions [34]. Changes in frequencies of clinically significant CBCL global scales (internalizing, externalizing, total problems) were assessed by means of the nonparametric McNemar test. To assess the ability of maternal stress at T1 to predict levels of CBCL/4–18 total scores at T2, a two-stage hierarchical multiple regression was calculated. If more than one targeted comparison was conducted per hypothesis, we applied Bonferroni-Holm corrections for type 1 error. Statistical significance was defined as *p* < 0.05 (two-tailed).

## 3. Results

Due to age restraints and missing data in some assessments, the sample sizes vary across analyses, as can be seen by the sample sizes reported in the tables.

### 3.1. Mental Health Problems of Subjects with 22q11.2DS (Hypothesis 1–3)

As 13 children had been younger than four years at T1, two different CBCL forms had been filled out by their mothers: the Child Behavior Checklist 1.5–5 [CBCL/1.5–5 [35,36] at T1, and the CBCL/4–18 at T2. In contrast, mothers of initially 4–14-year-old subjects had filled out the CBCL/4–18 both at T1 and T2. As the CBCL forms differ significantly with regard to item wording, number of items (CBCL/1.5–5: 100; CBCL/4–18: 113) and syndrome scales, results are presented separately for the following two groups: (i) initially 1.5–3-year-old children, and (ii) initially 4–14-year-old subjects with 22q11.2DS.

#### 3.1.1. Mental Health Problems of Initially 1.5–3-Year-Old Subjects with 22q11.2DS (*n* = 13)

With regard to CBCL/1.5–5 results at T1, a Mann–Whitney-U-test revealed no statistically significant differences between primary caregivers who only participated at T1 (*n* = 7) and primary caregivers who participated as well at T2 (*n* = 13). CBCL results for both study times are given in Table 1.

At T1, 4/13 subjects (30.8%), one boy and three girls, scored clinical on at least one CBCL/1.5–5 scale whereas twice as many subjects, three boys and five girls, were rated clinical on at least one CBCL/4–18 scale at T2. By means of Mann–Whitney U tests, no significant gender differences could be found for any of the CBCL scales at T1 and T2 which is contrary to our a priori hypothesis 2.

#### 3.1.2. Mental Health Problems among Initially 4–14-Year-Old Subjects with 22q11.2DS (*n* = 41; Missing: *n* = 1)

Regarding CBCL/4–18 results at T1, by means of paired-samples *t*-tests no statistically significant differences could be found between mothers who only participated at T1 (*n* = 28) and primary caregivers who participated as well at T2 (*n* = 41). For CBCL/4–18 results at T1 and T2 see Table 2.

One sample *t*-tests (one-sided) revealed significantly higher *t*-scores on all CBCL/4–18 scales at T1 and T2 than in the general population (T1: *p*s ≤ 0.002; T2: *p*s ≤ 0.001). Results were still significant after Bonferroni-Holm correction for multiple testing (see Table 2) thus confirming hypothesis 1. Effect sizes ranged from *d* = 0.43 (thought problems) to *d* =1.74 (social problems) at T1, and from *d* = 0.73 (delinquent behavior) to *d* = 2.11 (social problems) at T2.

Rates of clinical cases higher than 20 percent could be found for the following scales: social problems, internalizing problems and total problems at T1, and for social problems, attention problems and the three global scales at T2. Overall, total problems scales scored highest at T1 and T2 with regard to rates of clinical cases, and a comparison between clinical rates at T1 and T2 indicated an increase in clinical problems on almost all scales (exception: delinquent behavior).

With regard to our a priori hypothesis that boys scored significantly higher on the CBCL global scales compared to girls at T1 and T2, we found divergent results. As hypothesized, significantly higher scores could be found for internalizing problems (*t*(39) = 1.75, *p* = 0.045), externalizing problems (*t*(39) = 1.71, *p* = 0.047) and total problems (*t*(39) = 2.16, *p* = 0.019) at T1. However, only total problems were significantly higher in boys than in girls at T2 (*t*(39) = 1.86, *p* = 0.035). After Bonferroni-Holm correction no significant gender differences could be found for any of the CBCL global scales.

With the exception of delinquent behavior, significant increases of scores over time could be found by paired-samples *t*-tests for all CBCL/4–18 scales: withdrawn (*t*(39) = 2.42, *p* = 0.010), somatic complaints (*t*(39) = 1.94, *p* = 0.029), anxious/depressed (*t*(39) = 1.91, *p* = 0.032), social problems (*t*(39) = 1.94, *p* = 0.030), thought problems (*t*(39) = 2.98, *p* = 0.003), attention problems (*t*(39) = 3.08, *p* = 0.002), aggressive behavior (*t*(39) = 4.43, *p* < 0.001), internalizing problems (*t*(39) = 3.54, *p* < 0.001), externalizing problems (*t*(39) = 3.25, *p* = 0.001), total problems (*t*(39) = 3.10, *p* = 0.002). After Bonferroni-Holmes correction for multiple testing, significant increases could be found for the following scales (see Table 2): withdrawn, thought problems, attention problems, aggressive behavior, internalizing problems, externalizing problems, total problems. Effect sizes were mostly small, however the increase in internalizing problems reached a medium effect (see Table 2). Thus, our data confirm hypothesis 3.

#### 3.1.3. Overall Changes in Internalizing, Externalizing and Total Problems of Initially 1–14-Year-Old Subjects with 22q11.2DS (*n* = 54)

At T2, higher rates of clinical cases could be found for all CBCL global scales: total problems (T1: *n* = 19 [35.2%], T2: *n* = 30 [55.6%]), internalizing problems (T1: *n* = 14 [25.9%], T2: *n* = 21 [38.9%]), externalizing problems (T1: *n* = 8 [14.8%], T2: *n* = 14 [25.9%]). With regard to the CBCL global scales externalizing (*p* = 0.039) and total problems (*p* = 0.031), an exact McNemar’s test determined a statistically significant difference in the proportion of clinical to non-clinical cases between T1 and T2. Thus, these data in part confirm hypothesis 3.

### 3.2. Maternal Stress (Hypothesis 4–6)

With regard to maternal stress at T1, no statistically significant differences could be found between mothers who only participated at T1 and mothers who participated as well at T2.

As 11 subjects with 22q11.2DS were older than 14 years at T2, maternal stress could only be assessed in 44 mothers. Maternal stress was significantly lower than in the SOEBEK standardization study (*ps* after Bonferroni-Holm correction ≤ 0.029) both at T1 (*n* = 55; *M* = 40.84 [35th percentile], *SD* = 11.32, *d* = 0.483) and T2 (*n* = 44; *M* = 41.86 [40th percentile], *SD* = 11.45, *d* = 0.385).

No significant differences could be found between maternal stress at T1 (*n* = 44; *M* = 39.77 [30th percentile], *SD* = 11.52) and T2 (*n* = 44; *M* = 41.86 [40th percentile], *SD* = 11.45). Thus, our results did not confirm hypothesis 4. Moreover, no significant differences could be found with regard to maternal stress between mothers of girls and boys at T1 and T2.

At T2, six out of 42 subjects (14.3%; missing: *n* = 2) were suspicious of ASD according to the results of the German adaptation of the Autism Screening Questionnaire. 5 of them were male, and 2 out of 6 subjects had been classified by their mothers as intellectually disabled. As hypothesized a priori (hypothesis 5), significantly higher scores of maternal stress (*t*(40) = 1.95, *p* = 0.029, *d* = 0.874) could be found for these subjects at T2 when compared to subjects without such a suspicion.

At T2, 22 (9 females and 13 males) out of 50 subjects (44.0%; missing: *n* = 5) were classified by their mothers as intellectually disabled. Maternal stress was not significantly different between both groups. Thus, hypothesis 6 could not be confirmed.

### 3.3. Maternal Coping Strategies

Compared to the SOEBEK standardization study, one-sample *t*-tests with Bonferroni-Holm correction revealed significantly higher levels of use of social support in our study group both at T1 (*n* = 55; *M* = 21.80 [65th percentile], *SD* = 4.70, *p* = 0.009) and T2 (*n* = 44; *M* = 20.77 [60th percentile], *SD* = 3.78, *p* = 0.024). Coping strategies did not differ significantly between both study times (for detailed information see Table 3).

### 3.4. Maternal Personality Aspects: Strain and Satisfaction with Life (Hypothesis 7–8)

With regard to FPI-R subscales at T1, no statistically significant differences could be found between mothers who only participated at T1 and mothers who participated as well at T2.

Results of FPI-R scales at both study times are shown in Table 4.

As we had hypothesized a priori (hypothesis 7), we found significantly higher scores for maternal strain at T2 when compared to age and gender-matched subjects of the general population (*t*(54) = 2.20, *p* = 0.016, *d* = 0.28). However, in contrast to hypothesis 7, satisfaction with life was significantly lower in our study sample than in the general population (*t*(54) = 2.64, *p* = 0.011, *d* = 0.31). Moreover, no significant differences could be found for strain and satisfaction with life at T1 and T2 which is in part contrary to our a priori hypothesis 8.

### 3.5. Relationship between Child Behavior Problems, Maternal Stress and Strain (Hypothesis 9)

We had hypothesized a priori significantly positive relations between maternal stress and strain on the one hand and the CBCL global scales at both study times at the other hand. At T1, maternal stress correlated significantly with all global scales (Internalizing Problems: *r*(41) = 0.345, *p* = 0.027; Externalizing Problems: *r*(41) = 0.558, *p* < 0.001; Total Problems: *r*(41) = 0.574, *p* < 0.001), whereas we could not find any correlations between strain and the global scales. At T2, maternal stress was only positively correlated with the CBCL scales Externalizing Problems (*r*(44) = 0.599, *p* < 0.001) and Total Problems (*r*(44) = 0.589, *p* < 0.001), and strain showed only a significantly positive correlation with Total Problems (*r*(54) = 0.311, *p* < 0.011). After Bonferroni-Holm correction, correlations between maternal stress and the CBCL scales Externalizing Problems and Total Problems remained significant for both study times (*ps* = 0.005), whereas the correlation between strain and Total Problems at T2 was no longer significant. Correlations between maternal stress and the CBCL scales Externalizing Problems and Total Problems represented large effects. Thus, our a priori hypothesis could be confirmed in part.

### 3.6. Predictive Value of Maternal Stress at T1 for Child Behavior Problems at T2 (Hypothesis 10)

Although the sample size was quite small (*n* = 41), a two-stage hierarchical multiple regression was calculated to assess the ability of maternal stress at T1 to predict levels of CBCL/4–18 total scores at T2 after controlling for the influence of CBCL/4–18 total scores and maternal satisfaction with life at T1 as well as for the dichotomous classification of ASD suspicion (yes/no). Preliminary analyses were performed to ensure there was no violation of the assumption of normality, linearity, multicollinearity and homoscedasticity. The control variables were entered into Step 1 and explained 51.8% of the variance in CBCL/4–18 total scores at T2. After entry of the maternal stress at T1 values at Step 2 the total variance explained by the model was 52.9%, *F* [1,34] = 0.77, *p* = 0.368. This means that maternal stress at T1 explained only an additional 1.1% of variance in CBCL/4–18 total scores at T2, after controlling for CBCL/4–18 total scores and maternal satisfaction with life at T1 as well as for ASD suspicion. Thus, the final model did not prove to be of significance.

## 4. Discussion

The primary objective of this study was to examine longitudinal outcomes of behavior problems in young subjects with 22q11.2DS and their mothers’ stress, coping strategies, strain and satisfaction with life. In addition, we were interested to find out whether maternal stress might be a good predictor of emergent child behavior problems in the 22q11.2DS population. For this purpose, well-validated assessment instruments were applied. 57out of 94 (60.64%) of the addressed primary caregivers who had already participated in previous studies [16,17] returned filled-out questionnaires which is an acceptable result. 55 mothers could be included in our study which represents a sample size comparable to other longitudinal studies on psychosocial aspects in the 22q11.2DS population [13,20]. Moreover, mothers reported about physical and educational aspects of their children which were similar to those found in other studies (e.g., [13,20]).

### 4.1. Behavior Problems

With regard to psychological adjustment as assessed with the CBCL, our results clearly suggest an increase in behavior problems over time. Specifically, rates of externalizing and total problems in the clinical range showed an increase over time. Moreover, paired-samples *t*-tests revealed significantly higher behavior problem levels at T2 for the following CBCL/4–18 scales: withdrawn, thought problems, attention problems, aggressive behavior, internalizing problems, externalizing problems, and total problems. Effect sizes were mostly small, however, the increase in internalizing problems showed a medium effect size. Thus far, no longitudinal studies on children and adolescents with 22q11.2DS have reported similar results. Hooper and co-workers, the only group who also used the CBCL global scales to assess social-behavioral functioning [13], did not report about changes over a period of 3.5 years among the 32 subjects assessed at both study times. Other longitudinal studies (e.g., [20]) did not include behaviorally focused assessment instruments on more than one study time. The results of our study are in line with cross-sectional studies that have suggested an increase in behavior problems, especially internalizing problems, with age [16,17]. Specifically, a positive correlation between the subject’s age and the CBCL/4–18 scales withdrawn, anxious/depressed, thought problems, social problems, attention problems, internalizing problems and total problems as well as the CBCL/1.5–5 scale anxiety problems has been reported earlier [16,17].

Additionally, we found significantly higher behavior problem levels among children and adolescents with 22q11.2DS when compared to subjects from the general population. Specifically, initially 4–14-year-old subjects from our 22q11.2DS sample scored significantly higher on all CBCL/4–18 scales both at T1 and T2 when compared to the general population. These results are similar to the findings of earlier studies [13,15,16,20]. For example, Hooper et al. found significantly higher levels of internalizing and total problems on the CBCL compared to a control group [13], and Wagner et al. [20] reported significantly higher externalizing and internalizing composite scores on the parent rating scale of the Behavior Assessment System for Children (BASC-PRS [37]). As children with developmental delay or ID are about three times as likely to score in the clinical range for emotional and behavior problems than normally developing children [10,11,12], the high rate of clinical behavior problems is not surprising.

Contrary to our a priori hypothesis and findings from the general population [38,39,40,41], we could not find gender differences for any of the CBCL scales at T1 and T2 which is in line with the results of Swillen et al., 1999 [15]. Earlier studies on 22q11.2DS have drawn an unclear picture with regard to gender specific differences in child behaviors [15,16], and the reasons for this are unclear.

### 4.2. Maternal Stress and Coping

To the authors’ best knowledge, this is the first longitudinal study on maternal stress and coping strategies in the 22q11.2DS population. Compared to other parents of children with mental and/or physical disabilities, the mothers in our sample reported about significantly lower stress levels at both study times. However, effect sizes were only small. Similar results have been found in previous studies [16,17]. Additionally, significantly higher scores of maternal stress could be demonstrated at T2 for children who were suspicious of ASD (large effect) which is in accordance with the finding of a recent meta-analysis that overall levels of parenting stress are highest for parents of children with ASD and developmental delay compared to parents of children from other clinical groups [23]. Interestingly, we could not find a significant difference between parents of 22q11.2DS children with and without ID which might be due to the fact that this classification was based on maternal judgement, and not on objective information (i.e., IQ testing). Another reason might be, that the comparison group in this study, the SOEBEK standardization sample, did not comprise typically developing or healthy subjects which is in contrast to the PSI [26], which has been the primary measure of parenting stress in most other studies on this topic [23]. The PSI has been designed for use in the general population, and it includes questions that overlap with questions on parent-report measures of externalizing, but not internalizing behavior problems. In contrast, the SOEBEK has been specifically developed and validated for parents of children with mental and/or physical disabilities and does not include child behavior problems. Thus, it should allow to take better account of the special situation of parents of children with 22q11.2DS. Another reason for lower maternal stress levels might be that the children in this study were less disabled than the children in the standardization study of the SOEBEK. As significantly higher total parent stress scores on the PSI have been reported for parents of children and adolescents with 22q11.2DS compared to a community control [20], our results suggest that over all parenting stress in the 22q11.2DS population is higher than in the general population, but lower than among other parents of children with mental and/or physical disabilities. This information might be important both for professional counsellors and parents as it might be helpful for parental adaptation processes.

In our study, maternal stress was significantly positively associated with externalizing and total child behavior problems. Several earlier studies have well documented such an association across different clinical groups [23,42,43]. Moreover, Barroso and co-workers reported similarly large effect sizes as found in our study [23]. Despite the fact, that maternal stress was significantly positively associated with externalizing and total child behavior problems and that these behavior problems increased over time in our study sample we could not confirm a trend towards increasing maternal stress levels over time which is in contrast to previous cross-sectional studies in the 22q11.2DS population [16,17], but concordant with findings of a meta-analysis which could not identify child age as a significant moderator [23].

As child behavior problems are known to be the single most important predictor of caregiver’s psychological well-being [44] it seems that the mothers in our study sample were able to successfully cope with increasing behavior problems. In accordance with this hypothesis, we found levels of coping strategies similar to (partnership intensification, ability to meet own needs, focusing on the disabled child) or even significantly higher (use of social support) than among other parents of children with mental and/or physical disabilities, and these levels remained stable over time. As the rate of single parents was quite low in this study (12.7%), we suppose that partnership support has been an important aspect contributing to relatively low maternal stress levels. This hypothesis is supported by studies which showed that partnership support is the best predictor of coping for mothers of children with disabilities [45].

Some studies have shown that high levels of parenting stress contribute to a worsening in child behavior problems over time [e.g., 11,24]. Thus, we had hypothesized that maternal stress at T1 might have positive predictive value for child behavior problems as assessed with the CBCL/4–18 total problems scale at T2. A two-stage hierarchical regression did not confirm this hypothesis which, so far, has never been tested before in the 22q11.2DS population. In contrast, behavior problems at T1 seemed to be a much better predictor for subsequent problems at 4-years follow-up. However, it should be taken into account that our sample size was quite small (*n* = 41) for such a calculation.

### 4.3. Personality Aspects Strain and Satisfaction with Life

Thus far, no longitudinal studies have been done with regard to the personality aspects strain and satisfaction with life among mothers of children and adolescents with 22q11.2DS.

In this study, strain (the degree to which a person feels tense, overwrought or stressed) and satisfaction with life (the degree to which a subject is contented with life, optimistic or hopeful towards life) were significantly different compared to age and gender matched subjects of the general population (regardless of whether such a subject is a parent or not). Specifically, mothers reported increased levels of strain and reduced levels of satisfaction with life at T2. However, all effects were of small size. No significant correlations between strain and the CBCL/4–18 global scales could be demonstrated. While the results on strain are concordant with the findings of earlier studies [16,17], this does not apply to satisfaction with life. Specifically, previous studies have reported levels of satisfaction with life similar to age and gender matched subjects of the general population [16,17]. A possible reason for this discrepancy might be that our sample of mothers typically had to cope with challenges due to 22q11.2DS for a significantly longer time than parents participating in previous studies. However, no significant correlation of parental strain with the child’s age has been reported so far [16,17]. Moreover, we could not find any significant differences regarding strain and satisfaction with life between both study times indicating that there is no general development towards more strain and less satisfaction with life in mothers of children and adolescents with 22q11.2DS.

## 5. Limitations

Despite clear strengths, including the longitudinal design and carefully chosen clinically relevant measures, this study has some limitations, among them the lack of a matched control group. Generalizability of our results is further limited due to the fact that we used a convenience sample of participants. Thus, we cannot exclude a sample selection bias. Other reasons are the relatively low inclusion rate for this longitudinal study with a consecutively quite small number of subjects included (*n* = 55) causing limited statistical power for all analyses. As questionnaires were sent anonymously, we do not have any information about subjects whose mothers did not follow our request to participate in this study. Moreover, an increasing number of children and changes in economic situation might have reduced mothers’ time to answer questionnaires. However, there is no central register for 22q11.2DS in most countries, therefore questionable representativeness applies to many studies on 22q11.2DS.

There are some more limitations. First, there is a lack of objective information on subjects with 22q11.2DS, especially about their intelligence. As behavior problems increase with intellectual deficits [10,12], it would have been helpful to include results of intelligence tests. Second, we used only parent-report measures filled out by mothers. Father reports and self-report instruments might have added different perspectives and should therefore be included in further longitudinal studies. Third, we did not include a standardized measure of socioeconomic status. However, socioeconomic status has been found to predict social-behavioral outcomes in children with 22q11.2DS [46]. Fourth, we did not gather standardized information about maternal psychopathology (especially depression), the quality of parental relationship and parenting attitudes.

## 6. Conclusions

In sum, our results reveal that subjects with 22q11.2DS typically start early scoring significantly higher for various behavior problems than subjects of the general population, and this development seems to continue when they grow older. Specifically, internalizing problems showed an increase over time which represented a medium effect. Our results also indicate that maternal stress in the 22q11.2DS population is clearly associated with child behavior problems, especially externalizing problems. Moreover, various challenges due to 22q11.2DS seem to result in increased levels of strain and reduced levels of satisfaction with life among mothers of children and adolescents with 22q11.2DS. These findings are clinically absolutely relevant. They underline the importance of an early detection of the underlying genetic condition and suggest a growing need for professional consultation and interventions with increasing age of 22q11.2DS children and adolescents. Thus, clinical management should comprise careful monitoring as well as early and adequate interventions, if indicated, not only for somatic complaints but also for problems with psychological adjustment. For this purpose, a multidisciplinary approach is absolutely indicated. As parents’ perception of their children’s behaviors usually include an objective component, referring to actual child behaviors, and a subjective component, comprising parent-related factors, e.g., stress [47], it seems to be crucial to take parental stress into account when assessing child behavior by parent-report measures. Given the lack of studies on the effectiveness of parent management trainings, psychotherapeutic or pharmacological interventions in this special population, interventions, e.g., cognitive behavior therapy for internalizing disorders in children and adolescents, or stimulant medication for ADHD, should be chosen in accordance with guidelines of the psychological and psychiatric scientific societies. In order to promote coping, especially social support, contact to and support by national 22q11.2DS foundations can also be very helpful for many families.

## Figures and Tables

**Table 1:** Behavior problems of initially 1.5–3-year-old children at both study times (*n* = 13).

T1: CBCL/1.5–5			T2: CBCL/4–18		
Scale	Median *t*-Score (Range)	Clinical Cases (%)	Scale	Median *t*-Score (Range)	Clinical Cases (%)
Total Problems	50	3	Total Problems	64	7
	(29–75)	(23.1)		(48–79)	(53.8)
Internalizing Problems	53	3	Internalizing Problems	58	4
	(29–75)	(23.1)		(38–74)	(30.8)
Externalizing Problems	47	1	Externalizing Problems	61	4
	(26–67)	(7.7)		(44–71)	(30.8)
Anxious/ Depressed	52	1	Anxious/ Depressed	63	1
	(50°–70)	(7.7)		(50°–78)	(7.7)
Somatic Complaints	53	3	Somatic Complaints	50°	1
	(50°–72)	(23.1)		(50°–80 ^#^)	(7.7)
Withdrawn	56	1	Withdrawn	51	-
	(50°–82)	(7.7)		(50°–67)	
Attention Problems	53	1	Attention Problems	63	2
	(50°–70)	(7.7)		(50°–80 ^#^)	(15.4)
Aggressive Behavior	50°	-	Aggressive Behavior	60	1
	(50°–65)			(50°–74)	(7.7)
Emotionally Reactive	51	1	Social Problems	62	4
	(50°–83)	(7.7)		(50°–80 ^#^)	(30.8)
Sleep Problems	57	1	Thought Problems	50°	1
	(50°–76)	(7.7)		(50°–73)	(7.7)
			Delinquent Behavior	55	-
				(50°–66)	

Note: CBCL Child Behavior Checklist, ° T-value equal to or smaller than 50, ^#^ T-value equal to or higher than 80.

**Table 2:** CBCL/4–18 scales at T1 and T2: Comparison with general population and change over time (*n* = 41; missing: *n* = 1).

CBCL/4–18	T1 ^a^		T2 ^a^		Change over Time ^b^
Scale	Mean *t*-Score (*SD*; *d*)	Clinical Cases [*n*, (%)]	Mean *t*-Score (*SD*; *d*)	Clinical Cases [*n*, (%)]	*p*-Value (*d*)
Total Problems	60.71 *	16	64.07 *	23	0.016
	(8.13; 1.18)	(39.02)	(7.68; 1.58)	(56.10)	(0.42)
Internalizing Problems	56.68 *	11	61.51 *	17	0.006
	(10.16; 0.66)	(26.83)	(8.09; 1.27)	(41.46)	(0.53)
Externalizing Problems	55.20 *	6	58.00 *	11	0.009
	(8.04; 0.57)	(14.63)	(8.45; 0.86)	(26.83)	(0.34)
Withdrawn	58.51 *	5	61.90 *	6	0.05
	(8.76; 0.91)	(12.20)	(8.50; 1.28)	(14.63)	(0.39)
Somatic Complaints	55.95 *	1	58.63 *	4	n. s.
	(7.11; 0.69)	(2.44)	(8.11; 0.95)	(9.76)	(0.35)
Anxious/Depressed	57.68 *	2	60.17 *	5	n. s.
	(8.09; 0.84)	(4.88)	(8.43; 1.10)	(12.20)	(0.30)
Social Problems	65.90 *	11	68.41 *	15	n. s.
	(8.19; 1.74)	(26.83)	(7.24; 2.11)	(36.59)	(0.32)
Thought Problems	54.02 *	4	57.80 *	7	0.016
	(8.51; 0.43)	(9.76)	(9.50; 0.80)	(17.07)	(0.42)
Attention Problems	63.22 *	8	66.61 *	11	0.016
	(8.40; 1.43)	(19.51)	(7.78; 1.85)	(26.83)	(0.42)
Delinquent Behavior	55.02 *	1	55.95 *	0	n. s.
	(6.16; 0.60)	(2.44)	(5.72; 0.73)	(0)	(0.16)
Aggressive Behavior	56.54 *	2	59.54 *	4	0.006
	(7.35; 0.75)	(4.88)	(8.86; 1.01)	(9.76)	(0.37)

Note: ^a^: One-sample *t*-test with Bonferroni-Holm correction; ^b^: paired student *t*-test with Bonferroni-Holm correction; * *p* < 0.05; *SD*: Standard deviation; *d*: Cohen’s *d* (*M*_1_ − *M*_2_/σ_pooled_).

**Table 3:** Maternal coping strategies at T1 and T2 (*n* = 44).

SOEBEK	T1			T2		
Coping Scales	Mean; *SD* [Raw Score] (Mean Percentile)	Range [Raw Score] (Percentiles)	Clinical Cases [*n*, (%)]	Mean; *SD* [Raw Score] (Mean Percentile)	Range [Raw Score] (Percentiles)	Clinical Cases [*n*, (%)]
Partnership intensification	24.52; 7.48	6–36	5	24.52; 7.70	6–34	6
	(35th–40th)	(1st–99th)	(11.4%)	(35th–40th)	(1st–98th)	(13.6%)
Ability to meet own needs	17.75; 4.38	8–26	1	18.45; 4.41	8–27	1
	(50th)	[3rd–98th]	(2.3%)	(50th)	[3rd–98th]	(2.3%)
Use of social support	21.39; 4.14	9–29	1	20.77; 3.78	15–31	0
	(60th)	[3rd–98th]	(2.3%)	(60th)	[20th–99th]	
Focusing on disabled child	24.89; 4.95	13–33	0	25.09; 5.03	8–32	0
	(40th)	[<3rd–<95th]		(40th)	[<3rd–<95th]	

Note: *SD*: Standard deviation; SOEBEK: Social Orientation of Parents with Handicapped Children; T1: Time 1; T2: Time 2.

**Table 4:** FPI-R scales at T1 and T2: means, standard deviations, clinical cases (*n* = 55).

FPI-R Scales	T1		T2	
	Mean (Standard Deviation) [Stanine]	Clinical Cases [*n* (%)]	Mean (Standard Deviation) [Stanine]	Clinical Cases [*n* (%)]
Strain	5.31	6	5.60	4
	(2.21)	(10.9%)	(2.02)	
Life satisfaction	4.64	1	4.44	0
	(1.52)	(1.8%)	(1.58)	(0%)

## Data Availability

Not available.
